# Controlled Deposition of 3D Matrices to Direct Single Cell Functions

**DOI:** 10.1002/advs.202001066

**Published:** 2020-09-03

**Authors:** Sing Wan Wong, Stephen Lenzini, Raymond Bargi, Zhe Feng, Celine Macaraniag, James C. Lee, Zhangli Peng, Jae‐Won Shin

**Affiliations:** ^1^ Department of Pharmacology and Regenerative Medicine University of Illinois at Chicago Chicago IL 60612 USA; ^2^ Department of Bioengineering University of Illinois at Chicago Chicago IL 60607 USA; ^3^ Department of Aerospace and Mechanical Engineering University of Notre Dame Notre Dame IN 46556 USA

**Keywords:** droplet microfluidics, hydrogels, precision cell engineering, single cell engineering, stem cells

## Abstract

Advances in engineered hydrogels reveal how cells sense and respond to 3D biophysical cues. However, most studies rely on interfacing a population of cells in a tissue‐scale bulk hydrogel, an approach that overlooks the heterogeneity of local matrix deposition around individual cells. A droplet microfluidic technique to deposit a defined amount of 3D hydrogel matrices around single cells independently of material composition, elasticity, and stress relaxation times is developed. Mesenchymal stem cells (MSCs) undergo isotropic volume expansion more rapidly in thinner gels that present an Arg‐Gly‐Asp integrin ligand. Mathematical modeling and experiments show that MSCs experience higher membrane tension as they expand in thinner gels. Furthermore, thinner gels facilitate osteogenic differentiation of MSCs. By modulating ion channels, it is shown that isotropic volume expansion of single cells predicts intracellular tension and stem cell fate. The results suggest the utility of precise microscale gel deposition to control single cell functions.

## Graphical abstract

A droplet‐based microfluidic approach is developed to precisely control the amount of local 3D gel deposition around individual cells. Using this approach, this study shows that mesenchymal stromal cells expand in volume faster when they adhere to thinner gels, resulting in higher membrane tension and increased osteogenic differentiation independently of material composition and viscoelasticity.

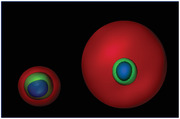

Cells utilize tactile mechanisms to physically probe the extracellular matrix.^[^ ^1^ ^]^ Advances in the design of engineered hydrogels have revealed that various matrix biophysical properties are sufficient to impact cellular functions independently of changes in biochemical cues, including matrix elasticity,^[^ ^2^, ^3^ ^]^ degradation,^[^ ^4^ ^]^ and stress relaxation.^[^ ^5^ ^]^ As a result, cells exert traction forces on matrices,^[^ ^6^, ^7^ ^]^ and subsequently tune their volume,^[^ ^8^, ^9^ ^]^ membrane and intracellular tension.^[^ ^10^ ^]^ These physical changes affect downstream biological functions, such as stem cell differentiation, via mechanosensitive transcription factors.^[^ ^11^ ^]^ Most studies with 3D hydrogels to date have revealed the importance of matrix properties in affecting cellular functions at the tissue scale by interfacing a population of cells with a bulk material. The prospect of utilizing the mechanical environment to program cell fate is attractive because it readily mimics the endogenous physiological situation, does not require additional chemical cocktails, and is readily scalable. Efforts to achieve physical control of cell fate via materials have generally involved engineering spatiotemporal mechanical control within hydrogels that interface with cells.^[^ ^12^ ^]^ For example, it was shown that spatial patterning of material stiffness affects stem cell fate.^[^ ^13^ ^]^ Additionally, dynamic control of material stiffness has been achieved in various systems,^[^ ^4^, ^14^ ^]^ which have shown to influence cellular functions. More recently, studies show that cells are highly sensitive to variations in local 3D matrix properties;^[^ ^15^ ^]^ however, precisely how the local matrix directly surrounding cells affects cell fate decisions remains unclear. Current approaches to interface a cell population with a bulk gel by uncontrolled mixing will likely lead to heterogeneity in the local amount of the gel presented to single cells, which makes cell–material interactions difficult to control and subsequently study. In addition, in order to tune cell‐to‐material volume ratios using a bulk material, it is often necessary to change cell population density, confounding the interpretation of whether observed biological effects are due to changes in cell–material or cell–cell interactions. Thus, alternative approaches are necessary to precisely control how much material is locally presented to each cell in a 3D space in order to study cell fate decisions driven by the local matrix environment.

Here, we report that a droplet‐based microfluidic approach can be developed to decouple the amount of hydrogel deposition around single cells from material composition and elasticity (**Figure** **1**A). Because CaCO_3_ nanoparticles are coated on cell surface, gelation of alginate only occurs in droplets that contain cells.^[^ ^16^ ^]^ By tuning the flow rates of aqueous and oil phases, the channel size of the microfluidic device, cell density in aqueous alginate solution, CaCO_3_ and acetic acid concentrations, we encapsulated single murine mesenchymal stem cells (MSCs) with varied gel deposition around cells (gel thickness: 2–15 µm, gel volume: 2000–45 000 µm^3^, total droplet size: 20–45 µm) (Figure 1B). The polymer concentration was kept at 1% w/v of ≈240 kDa alginate, and Young's modulus (*E*) was maintained at ≈2 kPa^[^ ^17^, ^18^ ^]^ (Figure 1C). Furthermore, stress relaxation times are not altered by gel deposition (Figure S1, Supporting Information). [Ca^2+^] in the medium remains physiological (≈2 × 10^−3^ m) across the different experimental groups (Figure S2A, Supporting Information). Crosslinking of the polymer occurs simultaneously with droplet formation, which helps maintain cell viability after encapsulation in the gel with varied deposition (Figure S2B, Supporting Information). The estimated swelling ratio (*Q*_v_) of gels remained the same (≈1.5) regardless of their size (Figure S2C, Supporting Information), which is expected for a constant w/v % and polymer crosslinking.^[^ ^19^ ^]^ Thus, this approach enables tunable local 3D gel deposition around single cells in a deterministic manner.

**Figure 1:**
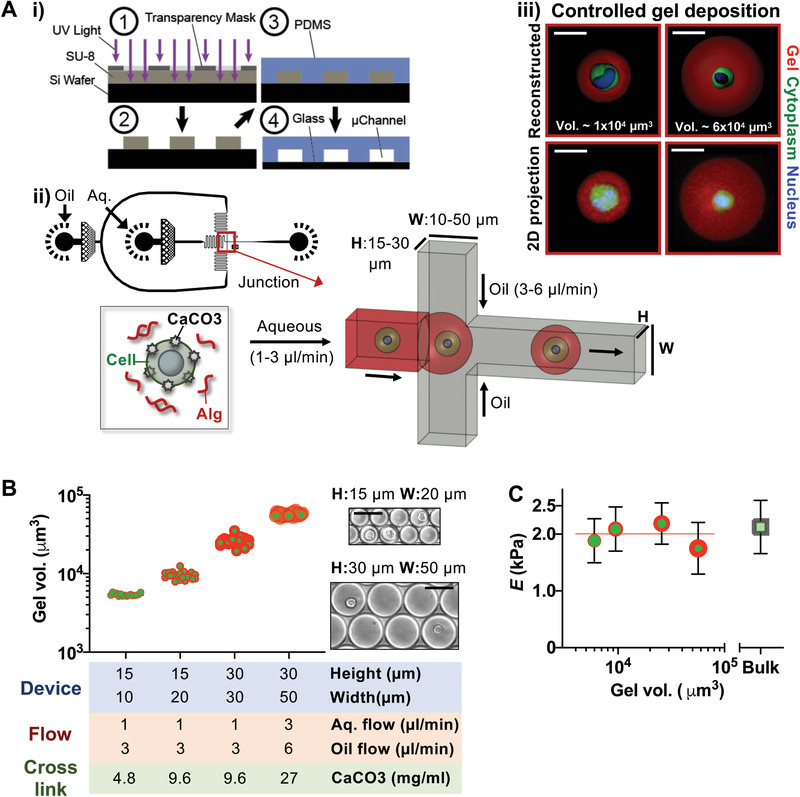
Controlled local 3D deposition of hydrogel matrices around single cells. A) A droplet microfluidic approach to control the local microscale deposition of alginate gels around single cells. i) Scheme illustrating the microfluidic device fabrication method (see the Experimental Section). (1 and 2) An SU‐8 mold with predefined patterns was fabricated by standard soft lithography with a digitally designed photomask. (3 and 4) The mold was used to create a PDMS device bonded to glass surface. ii) Overview of the droplet microfluidic device. The perspective view indicates the junction where the oil and the aqueous phases meet to form droplets. The aqueous phase consists of CaCO_3_‐coated cells and alginate precursors (alg.) dissolved in the buffered medium, while the oil phase consists of fluorinated oil, surfactant, and acetic acid. H: height, W: width. iii) Representative 3D‐reconstructed and 2D‐projected (maximum intensity) confocal images show MSCs encapsulated in varied gel deposition (1 vs 6 × 10^4^ µm^3^ in volume) ≈6 h after encapsulation. Red: alginate gel (alginate‐rhodamine), Green: cytoplasm (Calcein), Blue: nucleus (Hoechst) of MSCs. Scale bar = 20 µm. B) Local gel deposition can be precisely controlled by tuning device design, fluid flow parameters, and crosslinking, spanning over an order of magnitude in volume while maintaining tight control with coefficient of variation < 10% in all groups. Sample sizes for each device channel size (height, width in µm): (15h, 10w) *n* = 16, (15h, 20w) *n* = 23, (30h, 30w) *n* = 24, (30h, 50w) *n* = 25 from three independent experiments. Inset: representative images of gel droplets after generation. Scale bar = 50 µm. C) Young's modulus (*E*) of the gel remains constant at ≈2 kPa regardless of varied deposition as measured by AFM (Mean ± standard deviation (S.D.) from *n* = 3 independent experiments, 20 gels per experiment). *E* of the bulk alginate gel from the same material composition is shown as comparison (Mean ± S.D. from *n* = 3 gel preparations, 10 measurements per gel).

We sought to leverage the method to understand volume regulation of single cells as a function of local 3D matrix deposition, which remains an unaddressed fundamental question. MSCs were chosen as a model cell because they have been extensively investigated to understand cell–matrix interactions.^[^ ^2^, ^3^, ^4^, ^5^, ^6^, ^8^, ^9^, ^11^, ^16^, ^17^ ^]^ Clonally derived murine D1 MSCs were used, since they provide less cell‐to‐cell heterogeneity compared to primary cells.^[^ ^3^, ^5^, ^8^, ^9^ ^]^ Single MSCs were encapsulated with in the alginate gel with varied deposition: 9.6 (thin), 20.0 (medium), or 57.0 (thick) × 10^3^ µm^3^ in gel volume. The alginate gel‐coated MSCs were subsequently embedded in collagen‐I gel at a sparse density (5000 cells in 20 *μ*L) followed by confocal imaging analysis of live cells to evaluate their volume change over time. Gel‐coated MSCs were compared with MSCs encapsulated in a bulk alginate gel at the same cell density, composition, and *E* (Figure 1C). Molecular weight ≈240 kDa alginate was chosen, since single cells encapsulated in this gel formulation do not proliferate but remain viable in culture.^[^ ^16^ ^]^ Without any adhesion ligand, the volume of cytoplasm and nucleus is ≈1000 µm^3^ each, regardless of varied gel deposition (Figure S3A, Supporting Information). Uncoated MSCs embedded in collagen‐I within ≈2 h also show similar volume (Figure S3B, Supporting Information). Thus, we refer to 1000 µm^3^ as the baseline volume (*V*_0_) of cytoplasm and nucleus.

MSCs were then encapsulated in the alginate gel conjugated to the Arg‐Gly‐Asp (RGD) ligand, which binds to *α*_5_ *β*_1_ and *α*_v_ *β*_3_ integrins (alginate‐RGD). The volume of gel deposition remains unchanged over 3 d in culture (**Figure** **2**A). In contrast to the adhesion ligand‐free gel, MSCs in the thin alginate‐RGD gel rapidly (*t*_1/2_ = 1–2 h) undergo volume expansion to ≈1500 µm^3^ for both the cytoplasm (Figure 2B) and the nucleus (Figure 2C), while the rate of cell volume expansion becomes slower as local gel deposition increases. The rate of nuclear volume expansion is more sensitive to varied gel deposition than the rate of cytoplasmic volume expansion (Figure 2B,C insets). Similar effects were also observed with alginate conjugated to a CD44‐binding peptide (A5G27)^[^ ^20^ ^]^ (Figure S3C, Supporting Information), suggesting that the effects may be generalizable to other adhesion ligands. In contrast to cell volume expansion by cell spreading as observed in degradable^[^ ^4^ ^]^ or fast stress relaxing^[^ ^9^ ^]^ 3D gels, cell volume expansion in alginate‐RGD gels is isotropic, as MSCs remain mostly spherical over time (Figure S3D, Supporting Information). Nearly all MSCs remain within the gel over 3 d (Figure S3E, Supporting Information). The location of gel‐coated MSCs along the *z*‐depth (0–450 µm) does not impact cytoplasmic or nuclear volume regardless of varied gel deposition (Figure S3F, Supporting Information). Thus, tunable local 3D gel deposition with an adhesion ligand can be used to control the rate of isotropic single cell volume expansion.

**Figure 2:**
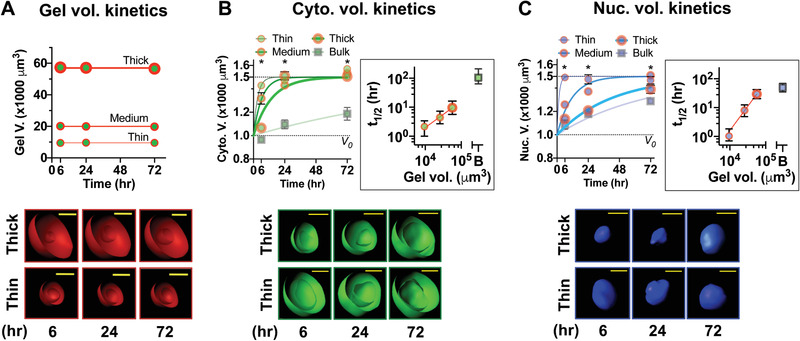
Local gel deposition tunes the rate of isotropic single cell volume expansion. A–C) Quantification of gel, cytoplasm, and nuclear volumes of MSCs over time in culture with varied gel deposition. For (A), the data were fit to a straight line with slope = 0, while for (B) and (C), the data were fit to a one‐phase association equation: *V* = *V*_0_ + (*V*_m_ − *V*_0_)(1 − e^−^ *^kx^*), where *V*_0_ = 1000 µm^3^ and *V*_m_ = 1500 µm^3^. All data are shown as mean ± standard error of the mean (S.E.M) from *n* = 3 independent experiments, 15 cells per experiment. *, B) *p* = 2.3 × 10^−6^, C) *p* = 6.4 × 10^−6^ for column factor (varied gel deposition) via two‐way ANOVA followed by Tukey's multiple comparisons test. The graphs in the inset of (B) and (C) show half‐maximum times (*t*_1/2_, in h) for each curve—the data from gel‐coated MSCs were fit to a power law equation *t*_1/2_ ∼ gel volume*^*α*^*, where *α* = 0.91 for (B) and 1.68 for (C), and error bars represent 95% confidence interval. “B” refers to bulk gels. The bottom panel for (A)–(C) shows representative confocal images from 3D reconstruction. Reconstructed images for the gel and the cytoplasm were sliced through the middle plane to show the inner surface. Scale bar for (A) = 20 µm and for (B) and (C) = 10 µm.

As cells expand in volume, they will likely stress the surrounding gel. Since cell volume expansion in engineered gel deposition is isotropic (Figure 2), it is possible to abstract the system into simple components and derive an analytical solution to calculate the stress on the inner gel surface (*σ*_gel_) with a given *E* when an encapsulated cell with the radius *r*_1_ expands radially by *u*_0_ in response to an adhesion ligand (**Figure** **3**A i). The analytical solution of the corresponding linear elasticity problem (Supporting Information) suggests that when the gel is incompressible (Poisson's ratio, *ν* = 0.5), *σ*_gel_ can be expressed as a function of the gel thickness (*d*_gel_)

(1) $$\begin{array}{c} \sigma_{\mathrm{gel}}=\frac{Eu_{0}}{r_{1}}\;\left(\frac{4r_{1}^{3}}{3{\left(r_{1}+d_{\mathrm{gel}}\right)}^{3}}+\frac{2}{3}\right) \end{array}$$

**Figure 3:**
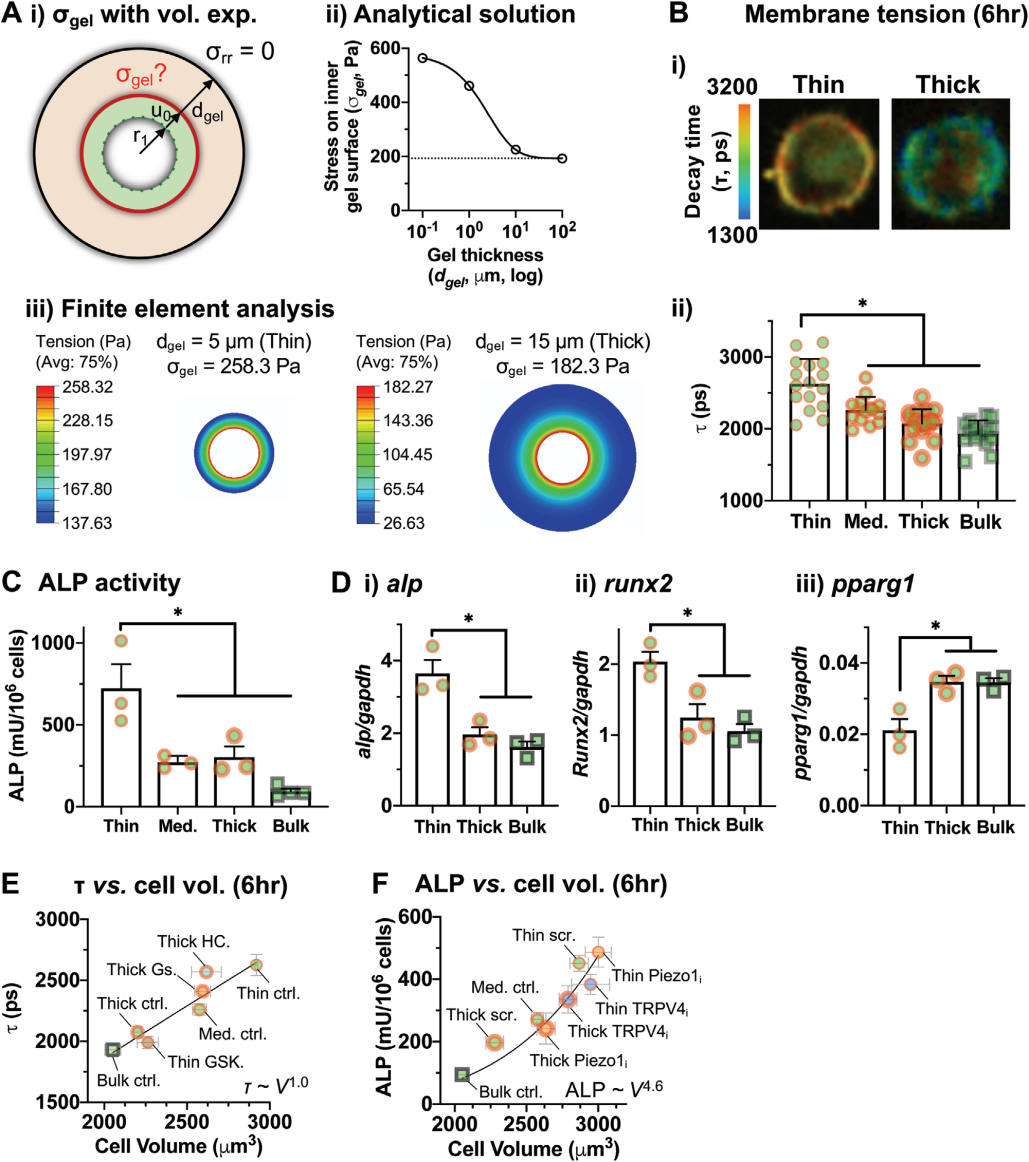
Isotropic volume expansion of single cells modulated by varied gel deposition predicts intracellular tension and stem cell differentiation. A) Calculation of the stress on the gel upon isotropic cell volume expansion. i) Schematic of a simple system where a cell with the radius *r*_1_ expands by the length *u*_0_ against the gel with varied thickness (*d*_gel_), leading to the stress on the inner gel surface (*σ*_gel_). External stress is set to zero (*σ*_rr_ = 0). ii) Analytical solution for *σ*_gel_ when *E* = 2000 Pa, *r*_1_ = 7.82 µm (radius of MSCs prior to volume expansion), and *u*_0_ = 1.13 µm (cell volume expansion by 50%) (Supporting Information). iii) Finite element analysis showing a gradient of tension along the gel depth when a cell expands in volume by 50%. B) Measurement of membrane tension at 6 h after encapsulation of MSCs in alginate‐RGD gels. Fluorescence lifetime imaging (FLIM) was used to evaluate decay lifetime (*τ*) of a lipid tension reporter that binds to cell membrane. i) Representative images showing *τ* when MSCs are in the thin or thick gel. ii) *τ* values from MSCs in gels with varied deposition. *n* = 15 cells pooled from three independent experiments, and shown as mean ± S.D. *, *p* = 1.5 × 10^−9^ via one‐way Welch's ANOVA followed by Dunnett T3 multiple comparisons test. C) Quantification of alkaline phosphatase (ALP) activity with varied gel deposition after 7 d culture of MSCs in the osteogenic medium. *n* = 3 independent experiments for each gel deposition and *n* = 4 for the bulk gel. D) Gene expression of i) *alp*, ii) *runx2*, and iii) *pparg1* after 10 d culture in the mixed osteogenic and adipogenic medium. *n* = 3 independent experiments. For (C) and (D), mean ± S.E.M; *, (C) *p* = 0.0013, (D) i) *p* = 0.0034, ii) *p* = 0.0076, iii) *p* = 0.0071 via ordinary one‐way ANOVA followed by Tukey's multiple comparisons test. E) Correlation analyses of membrane tension (*τ*) versus cell volume. Ctrl: control, HC: HC‐067047, Gs: GsMTx‐4, GSK: GSK1016790A. F) ALP activity versus cell volume. Scr: scrambled, Piezo1_i_: Piezo1 siRNA, TRPV4_i_: TRPV4 siRNA. For both (E) and (F), cell volume at 6 h after encapsulation is shown. The data were fit to a power‐law equation and shown as mean ± S.E.M.

Given the constant *E*, *r*_1_, and *u*_0_, *σ*_gel_ is increased with thinner *d*_gel_ (Figure 3A ii)—this trend is also observed if the gel is extendable (i.e., 0 ≤ *ν* ≤ 0.5). The analysis further shows that if the gel is extendable, the gel volume is expected to increase as a result of cell volume expansion, and hence the *E* of the gel will likely decrease due to reduced polymer density (Supporting Information). However, the atomic force microscopy (AFM) analysis shows that *E* of the outer gel surface remains unchanged when MSCs are encapsulated in the thin gel in the presence or absence of RGD—with or without cell volume expansion, respectively (Figure S4A, Supporting Information). Hence, the gel consisting of 1% w/v, 240 kDa alginate as used in this study is likely close to incompressible. Large strain finite element analysis yields similar results as the analytical solution, and also shows that the stress on the gel is the highest at the cell–matrix interface (Figure 3A iii; Supporting Information). Thus, the gel thickness is an important determinant of the gel stress exerted during isotropic cell volume expansion.

To test whether cells interpret differences in gel stress and subsequently tune their membrane tension as a function of varied gel deposition, we leveraged a fluorescent lipid tension reporter that changes fluorescent lifetime (*τ*) upon molecular twisting in response to cell membrane tension^[^ ^21^ ^]^ as measured by two‐photon fluorescence lifetime imaging (FLIM) (Figure S4B, Supporting Information). Since the reporter is a small molecule, it readily diffuses into alginate gels, which are ≈5 nm in pore size,^[^ ^22^ ^]^ and labels the membrane of encapsulated MSCs (Figure 3B i). As a positive control, the relationship between cortical tension measured by evaluating *E* using AFM and membrane tension measured by *τ* after seeding cells on 2D poly(ethylene glycol)diacrylate (PEGDA) hydrogels conjugated to RGD with varied elasticity. As expected, both *E* (Figure S4Ci, Supporting Information) and *τ* (Figure S4Cii, Supporting Information) of cells increase when substrate elasticity increases—plotting these two parameters shows that *E* scales with *τ* with power law exponent (*α*) ≈ 1.8 (Figure S4Ciii, Supporting Information). Results show that MSCs in the thin alginate‐RGD gel show significantly higher *τ* than MSCs in thicker gels (Figure 3B ii). Thus, tunable local 3D gel deposition with an adhesion ligand can be used to control the membrane tension of single cells independently of local matrix *E*.

Greater cell volume expansion^[^ ^9^ ^]^ or intracellular tension^[^ ^2^, ^4^, ^8^ ^]^ has been reported to skew commitment of MSCs toward osteogenic lineages. Since both phenotypes are observed with varied gel deposition, we tested whether tuning microscale gel deposition alone is sufficient to influence MSC differentiation. After culturing alginate‐RGD gel‐encapsulated MSCs for 7 d in the medium containing an osteogenesis‐promoting cocktail, alkaline phosphatase (ALP) activity was measured to quantify early osteogenic commitment. Strikingly, ALP activity increases as gel deposition becomes thinner even when the gel *E* remains at ≈2 kPa (Figure 3C). To test whether these results reflect osteogenic commitment of multipotent MSCs, gel‐coated MSCs were cultured for 10 d in the presence of both osteogenesis and adipogenesis‐promoting cocktails. While MSCs in the thin gel show higher gene expression levels of osteogenic markers, including *alp* and *runx2*, MSCs in the thick gel and the bulk gel show a higher level of an adipogenic marker, *pparg1* (Figure 3D). The diffusivity of small molecules that promote MSC differentiation is less likely impacted by varied gel deposition, since the diffusion kinetics of fluorescein (FITC)‐dextran (≈20 kDa) into the gel remains unchanged (Figure S5, Supporting Information). Thus, varied local 3D gel deposition with an adhesion ligand impacts the lineage specification of single MSCs.

To establish the causality between isotropic cell volume expansion and membrane tension or osteogenic differentiation regulated by varied local gel deposition, we modulated the activity of mechanosensitive ion channels, including Piezo1 and transient receptor potential vanilloid 4 (TRPV4), since they play roles in cell volume regulation.^[^ ^23^ ^]^ Activation of some ion channels is known to drive cell shrinkage by water efflux.^[^ ^24^ ^]^ Treatment of MSCs in the thin alginate‐RGD gel with GSK1016790A (GSK101, TRPV4‐selective agonist) for 2 h after encapsulation reduces both cytoplasmic and nuclear volumes (Figure S6A, Supporting Information). In contrast, treatment of MSCs in the thick gel with GsMTx‐4 (inhibitor of some mechanosensitive ion channels, including the Piezo family) or HC‐067047 (selective TRPV4 inhibitor) increases both cytoplasmic and nuclear volumes (Figure S6B, Supporting Information). As expected, GSK101 reduces membrane tension in the thin gel (Figure S6C, Supporting Information), while GsMTx‐4 or HC‐067047 increases membrane tension in the thick gel (Figure S6D, Supporting Information). Consequently, membrane tension directly correlates with cell volume (Figure 3E).

To reduce any potential nonspecific effects by prolonged treatment of ion channel modulators during MSC differentiation, MSCs were treated with small interfering RNA (siRNA) against Piezo1 or TRPV4 prior to encapsulation, which leads to a ≈70% decrease in target gene expression (Figure S7A, Supporting Information). The knockdown of either Piezo1 or TRPV4 does not impact cell volume in the thin alginate‐RGD gel compared to the scrambled control (Figure S7B, Supporting Information). While the knockdown of TRPV4 accelerates the expansion of both cytoplasmic and nuclear volumes in the thick gel, the knockdown of Piezo1 fails to increase nuclear volume (Figure S7C, Supporting Information), suggesting that Piezo1 and TRPV4 distinctly impact volume expansion of MSCs in local gel deposition. As expected, the knockdown of either Piezo1 or TRPV4 does not impact ALP activity in the thin gel (Figure S7D, Supporting Information). While Piezo1 siRNA fails to rescue ALP activity, TRPV4 siRNA rescues ALP activity in the thick gel (Figure S7E, Supporting Information), suggesting that isotropic volume expansion of both the cytoplasm and the nucleus will likely be required to promote osteogenic differentiation. The results collectively show that ALP activity scales with cell volume with the power low exponent *α* ≈ 4.6 (Figure 3F).

Together, we describe a method to control the microscale deposition of engineered hydrogels around individual cells in a 3D space. We show that varied gel deposition alone has a profound impact on the rate of isotropic cell volume expansion in the presence of an adhesion ligand, which subsequently regulates membrane tension and stem cell differentiation in a predictable manner. Results from this study will help facilitate precision engineering of cell–material interactions for fundamental biological science and translational therapeutic applications. For example, the method described here can be readily expanded to elucidate downstream mechanisms behind how single cells respond to engineered local matrix deposition, and how gene expression governing cell fate decision and long‐term lineage differentiation is subsequently altered in a distinct manner from elastic modulus and viscoelasticity. Our method can also be adapted to be combined with single cell sequencing technologies, in order to understand single cell heterogeneity in biophysical cell–matrix interactions. In engineering cells for regeneration of rigid tissues such as bone, our findings suggest a practical strategy to augment the osteogenic potential of donor MSCs by using a minimal amount of materials, potentially reducing the risk of foreign body reaction and the cost of materials.

## Experimental Section

##### Cell Culture

Clonally derived D1 mouse MSCs were purchased from American Type Cell Culture (CRL‐12424, ATCC). D1 MSCs were cultured in complete medium composed of high‐glucose Dulbecco's modified Eagle medium (DMEM; Thermo Fisher Scientific) supplemented with 10% fetal bovine serum (Atlanta Biologicals), 1% penicillin‐streptomycin (P/S), and 1% GlutaMAX (Thermo Fisher Scientific). Cells were passaged when they reached ≈80% confluence by detaching with trypsin‐EDTA (Thermo Fisher Scientific). D1 MSCs with passage number less than 13 were used in the study.

##### Alginate Preparation

Sodium alginate with ≈240 kDa molecular weight (LF200) was purchased from FMC Biopolymer. To enable cell adhesion to alginate, an integrin‐binding peptide consisting of Arg‐Gly‐Asp (GGGGRGDSP; Peptide 2.0) or a CD44‐binding peptide A5G27 (RLVSYNGIIFFLK; Peptide 2.0) was covalently conjugated to alginate by 1‐ethyl‐dimethylaminopropyl (EDC) and N‐hydroxy sulfosuccinimide (NHS) (Sigma) chemistry with a degree of substitution of ≈20 as described previously.^[^ ^25^ ^]^ After conjugation, alginate was dialyzed against decreasing concentrations of NaCl, charcoal‐treated, filter‐sterilized, and lyophilized. Lyophilized alginate was stored in −20 °C and dissolved in DMEM within one week prior to experiments. To visualize alginate gels, a small amount (final w/v = 0.05%) of 10/60 alginate (≈120 kDa; FMC Biopolymer) coupled with Lissamine rhodamine B ethylenediamine (Thermo Fisher Scientific) was added prior to gel formation.

##### Microfluidic Device Fabrication

Microfluidic devices were fabricated using soft lithography as described previously.^[^ ^26^ ^]^ To develop a photoresist, SU‐8 3025 (MicroChem) was deposited onto a silica wafer to a defined height, and cured by UV light exposure through a transparency mask (CAD/Art Services) for patterning. Polydimethylsiloxane (PDMS) (Dow Corning) was then mixed with cross‐linker at ratio 10:1, degassed, poured, and cured for at least 3 h at 65 °C. The cured PDMS was peeled off the wafer and bonded to a glass slide by oxygen‐plasma treatment of both surfaces. Microfluidic channels were then treated with Aquapel (PPG Industries) and dried. Polyethylene tubing (inner diameter: 0.38 mm; outer diameter 1.09 mm) and 27G × 1/2 needles were used to connect microfluidic channels to syringes (Becton Dickinson). Aqueous and oil flow rates in syringes were controlled by syringe pumps (Harvard Apparatus).

##### Tuning Alginate Gel Deposition around Single Cells

CaCO_3_ nanoparticles (CalEssence; 900 nm diameter) were resuspended in complete medium and dispersed by sonication with Vibra Cell Sonicator at 75% amplitude for 1 min. The nanoparticles were then centrifuged at 50 *g* for 5 min to discard larger aggregates, followed by 1000 *g* for 5 min for collection. Purified CaCO_3_ nanoparticles were resuspended with serum‐free DMEM medium—the concentration of CaCO_3_ was increased from 4.8 to 27.0 mg mL^−1^ with thicker alginate gel deposition. Cells were then incubated with CaCO_3_ by rotation at room temperature for 1 h. Excess CaCO_3_ nanoparticles were then washed out by centrifugation. The aqueous phase was prepared by resuspending CaCO_3_‐coated cells in the buffer consisting of DMEM with 50 × 10^−3^ m HEPES, 10% FBS, 1% P/S at pH 7.4, and mixing cells with 1% w/v alginate solution. The oil phase consisted of fluorinated oil (HFE‐7500; 3M) with 1% perfluoropolyether (PFPE, Krytox; Miller Stephenson) as a surfactant and 0.03% acetic acid as an initiator of Ca^2+^ release from CaCO_3_. The aqueous and oil phases were injected into the microfluidic device. For thicker alginate gel deposition, channel dimensions of the microfluidic device and flow rates were increased as noted in Figure 1B. Emulsion was collected every 20 min followed by 40 min rotation at room temperature. Emulsion was then broken by the addition of 10% 1H, 1H, 2H, 2H‐perfluoroctanol (Alfa Aesar). Gel‐coated cells were washed twice with serum‐free DMEM. Roughly 12 000 gel‐coated cells were embedded in 50 µL of 1.25 mg mL^−1^ collagen‐I matrix (Rat tail, Gibco/Thermo Fisher Scientific) on a 48‐well glass bottom plate (P48G‐1.5‐6‐F; MatTek Corporation), followed by culture at 37 °C in complete DMEM.

##### Cell Encapsulation in Bulk Alginate Hydrogels

Cells were resuspended in 1% w/v LF200 alginate in DMEM, and rapidly mixed with calcium sulfate by syringes. A final concentration of 10 × 10^−3^ m calcium sulfate was used to form the bulk hydrogel with *E* ≈ 2 kPa. The mixed solution was deposited between two glass plates with a 1 mm void thickness. After 1.5 h, hydrogels were punched into discs and cultured in a 96‐well glass bottom plate (P96G‐1.5‐5‐F, MekTak) in complete medium.

##### Mechanical Analysis of Gel Deposition around Cells

Gel‐coated cells were immobilized on a glass slide precoated with 0.1 mg mL^−1^ of poly‐l‐lysine for 2 h. The slide was then placed in an MFP‐3D system (Asylum Research) to perform AFM with a silicon nitride cantilever with an 18° pyramid tip (MLCT, Bruker). A spring constant of the cantilever was determined from thermal fluctuations at room temperature (20–40 mN m^−1^) before each analysis. A fluorescent microscope was used to bring the cantilever to the gel surface. Indentation was then performed under contact mode with force distance 500 nm and 1 µm s^−1^ velocity until the trigger cantilever deflection voltage (0.5 V) was reached, followed by retraction. To calculate Young's modulus (*E*), force–indentation curves were fitted to the Hertzian model with a pyramid indenter^[^ ^27^, ^28^ ^]^ and Poisson's ratio (*ν*) = 0.5.

Stress relaxation times of gels with varied deposition were measured by AFM as previously described.^[^ ^27^, ^28^ ^]^ The cantilever was brought toward the gel surface at velocity 1 µm s^−1^. Once the trigger cantilever deflection voltage (0.5 V) was reached, *z*‐height was increased to 100 nm, followed by a 9 s dwell time, and then the cantilever was retracted. The deflection of the tip during the dwell period was recorded under a constant load with the sampling rate = 120 Hz. Values for tip deflection over the dwell period were converted to force using the spring constant measured during AFM calibration. For all time *t*, force (*F*) was converted to stress (*S*) using the equation for pyramidal tip geometry^[^ ^28^ ^]^

(2) $$\begin{array}{c} S\left(t\right)=\frac{F\left(t\right)\sqrt{2}\left(1-\nu \right)}{\gamma_{0}^{2}\tan\left(\alpha \right)} \end{array}$$

where *ν* is Poisson's ratio (assumed to be 0.5 for hydrogels), *γ*_0_ is the constant strain over the dwell period, and *α* is the pyramidal face angle. Stress curves over the dwell period were then fit to the equation

(3) $$\begin{array}{c} S\;\left(t\right)=E_{R}\;\left(1+\frac{\tau_{\sigma }-\tau_{\varepsilon }}{\tau_{\varepsilon }}e^{-\frac{t}{\tau_{\varepsilon }}}\right) \end{array}$$

as described previously,^[^ ^27^ ^]^ where *E*_R_ is the relaxed modulus, *τ*_*σ*_ is the time of relaxation of deformation under constant load, and *τ*_*ε*_ is the time of relaxation of load under constant deformation. *E*_R_ can be calculated from Young's modulus from force–indentation curves, since Young's modulus = 1.5*E*_R_.^[^ ^27^ ^]^

##### Confocal Imaging and Image Analysis

Cells in gels containing alginate‐rhodamine were incubated with 1 × 10^−6^ m of Hoechst 33342 and 2 × 10^−6^ m of calcein AM (both from Thermo Fisher Scientific) for 1 h to stain nucleus and cytoplasm, respectively. Samples were then washed with HBSS and maintained in Fluorobrite DMEM (Thermo Fisher Scientific) at 37 °C 5% CO_2_ during confocal imaging in the Zeiss LSM 770 system with a motorized stage and the 20×/0.8 M27 Plan‐Apochromat objective. To analyze cell volume, *z*‐stacks were captured with 60–90 µm total depth with each image at 0.77 µm for 75–115 images per *z*‐stack. The stacks were then analyzed in Imaris (Bitplane, version 7.7.2). 3D reconstruction of each stack was performed by the built‐in algorithm. Voxels were generated for red (alginate‐rhodamine), green (calcein), and blue (Hoechst) signals after automatic thresholding. Thresholding values varied less than 10% across all the images from different experiments. A gel‐coated cell was considered an outlier and hence excluded from the analysis if it met one of the following criteria: 1) Blue voxels extend beyond the boundary of green voxels. 2) Green voxels extend beyond the boundary of red voxels. 3) Red voxels do not contain green or blue voxels inside. 4) Green and blue voxels are not within red voxels. The total voxels above the threshold were then calculated to quantify gel, cytoplasmic, and nuclear volumes of each gel‐coated cell. Sphericity of gel, cell, and nucleus was analyzed from the same set of voxels and defined as (*π* ^1/3^(6*V*)^2/3^)/*A*, where *V* is volume and *A* is surface area.

##### Chemical Inhibitors

The following chemical inhibitors were purchased from Cayman Chemical: GSK1016790A (No. 17289) and HC‐067047 (No. 20927). GsMTx‐4 was purchased from Alomone Labs (No. STG‐100).

##### Measurement of Extracellular Ca^2+^ Concentration

The calcium assay kit (Cayman Chemical) was used to evaluate Ca^2+^ concentration in the culture medium according to the manufacturer's protocol, based on a colorimetric reaction between *o*‐cresolphthalein and calcium. The absorbance of the purple color was measured at 575 nm.

##### Finite Element Analysis to Model Gel Stress

In the case of large deformation with rubber‐like elasticity, a nonlinear finite element method was applied to solve the boundary value problem using the commercial finite element package Abaqus. The axisymmetric formulation was used to solve the 3D problem. ≈2000 quadrilateral axisymmetric elements were used to reach convergence. The displacement boundary condition (*u* = *u*_0_) was applied at the inner boundary, and the stress‐free boundary condition (*σ*_rr_(*r* = *r*_1_ + *d*_gel_) = 0) was applied at the outer boundary. The incompressible neo‐Hookean material was used to consider the rubber‐like elasticity of the gel. The strain energy potential of neo‐Hookean material is given as $U\;=\frac{G}{2}\;\left(I_{1}-3\right)$, where $I_{1}=\lambda_{1}^{2}\;+\lambda_{2}^{2}+\lambda_{3}^{2}$ is the first invariant of deformation, *λ*_1_ *, λ*_2_ *, λ*_3_ are the principal stretches, and *G* is the shear modulus (Supporting Information). Stress fields were then visualized using the ABAQUS CAE postprocessing interface.

##### Measurement of Membrane Tension by Fluorescent Lifetime Imaging Microscopy (FLIM)

Cells in gels were incubated with 1 × 10^−6^ m of Flipper‐TR lipid membrane tension probe (Cytoskeleton, Inc.)^[^ ^21^ ^]^ in complete medium for 30 min. FLIM was performed in the Ultima Multiphoton Microscope System equipped with a Becker and Hickl time‐correlated single‐photon counting module (Bruker). The probe was excited at 920 nm by the Chameleon Ultra II Two‐Photon laser operating at 80 MHz. The emission signal was collected through a bandpass 595/50 nm filter for 1 min. Signal decay time (*τ*) values were extracted by fitting the average photon count versus time graph to a two‐phase exponential decay fit (Figure S4B, Supporting Information) in the data analysis software SPCimage (Becker & Hickl GmbH)—*τ* values correspond to the first component of the lifetime (*τ*_1_) in the curve fit, since the second component accounts for a minority of the signal.

##### Retrieval of Cells from Gels

Cells in gels were retrieved by digesting with 2.5 mg mL^−1^ collagenase P (Sigma), 4 mg mL^−1^ alginate lyase (Sigma), and 0.125% trypsin‐EDTA (Thermo Fisher Scientific) at 37 °C for 30 min. Samples were then centrifuged at 3000 rpm for 5 min and washed twice with HBSS, followed by downstream analyses.

##### Cell Viability Analysis by Flow Cytometry

Cells retrieved from gels were added to the stain buffer consisting of HBSS with 2 × 10^−6^ m of calcein AM (Biotium) and 2 × 10^−6^ m ethidium homodimer‐1 (Thermo Fisher Scientific) for 30 min. Samples were then analyzed by flow cytometry using LSRFortessa (Becton Dickinson). An event threshold of 5000 in forward scatter was used to exclude debris. Percent cell viability was calculated by dividing the number of calcein^+^ ethidium^−^ events by the total event number. In some cases, APC beads (Calibrite; Becton Dickinson) with a known number were added in each sample to calculate an absolute number of viable and dead cells.

##### Diffusion Assay

To characterize the diffusion kinetics with varied gel deposition, small (≈25 µm in diameter) or large (≈45 µm in diameter) gels without cells were generated by mixing alginate with 4.8 mg mL^−1^ CaCO_3_ and running through the droplet microfluidic device by using the same parameters as single cell encapsulation (Figure 1A), followed by confirmation of Young's modulus by AFM (*E* ≈ 2 kPa). Small, large, and bulk alginate gels were then incubated with fluorescein isothiocyanate‐dextran (FITC‐dextran) with average molecular weight ≈20 kDa (Sigma). The media were collected, and gels were digested after incubation by using the cell retrieval protocol at different time points: 30, 60, 120, and 1440 min. FITC‐dextran in media and digested gels were then measured in a black 96‐well plate at excitation/emission = 490/520 nm by using PHERAstar (Version 5.41).

##### MSC Differentiation and Alkaline Phosphatase Activity Assay

To evaluate the differentiation potential of MSCs in gels 1 d after encapsulation, they were cultured in medium supplemented with either an osteogenic chemical cocktail (No. CCM009) alone or both osteogenic and adipogenic (No. CCM011) cocktails for 7 or 10 d, respectively. All reagents for MSC differentiation were purchased from R&D Systems. One half of each sample was used to quantify an absolute number of viable cells by flow cytometry as described previously, while the other half was used to evaluate ALP activity. To quantify ALP activity, samples were lysed with 100 *μ*L passive buffer (No. E1941, Promega) for at least 10 min at 4 °C. Each lysate was then added to a black 96‐well plate preloaded with 100 *μ*L 4‐methylbelliferyl phosphate (4‐MUP) substrate (No. M3168, Sigma). Signals were acquired with excitation at 360 nm and emission at 450 nm using a plate reader. Recombinant mouse ALP protein (Novus Biologicals) was used to generate a standard curve for calibration. ALP activity of each sample was then normalized to the number of viable cells.

##### Gene Expression Analysis

Cells were lysed with 1 mL of Trizol reagent (Thermo Fisher Scientific) for 10 min. Samples in Trizol were stored at −80 °C if not processed immediately up to one week. 200 µL of chloroform was added per mL Trizol for phase separation. Samples were centrifuged for 15 min at 12 500 rpm, 4 °C. The top layer containing RNA was collected into a new tube, and then precipitated with 250 µL isopropanol, and 250 µL 0.8 m sodium citrate combined with 1.2 m sodium chloride for at least 15 min at 4 °C. Samples were then centrifuged at 12 500 rpm for 15 min at 4 °C. The supernatant was removed, and the precipitated RNA was washed with 75% ethanol, followed by centrifugation for 5 min at 7500 rpm, 4 °C. After removing ethanol, purified RNA was resuspended in 15 µL of RNase‐free water (Thermo Fisher Scientific). NanoDrop spectrophotometer (Thermo Fisher Scientific) was used to quantify RNA concentration and quality. cDNA was obtained by reverse transcription using SuperScript‐III reverse transcriptase (Thermo Fisher Scientific). For each sample, 50 ng cDNA was added to each well in triplicate, followed by the Power SYBR Green PCR Master Mix (Applied Biosystems). Quantitative PCR was performed in the ViiA7 qPCR system (Thermo Fisher Scientific). Relative gene expression was calculated using the ΔΔCt method by comparing each cycle threshold (Ct) value to the reference gene (*gapdh*). Primer sequences are described as follows:

**Table:** 

*gapdh* *(NM_001289726.1)*	F: CTTTGTCAAGCTCATTTCCTGG R: TCTTGCTCAGTGTCCTTGC
*alp* *(NM_001287172.1)*	F: CTCCAAAAGCTCAACACCAATG R: ATTTGTCCATCTCCAGCCG
*runx2* *(NM_001146038.2)*	F: GCTATTAAAGTGACAGTGGACGG R: GGCGATCAGAGAACAAACTAGG
*pparg1* *(NM_001127330.2)*	F: TGTTATGGGTGAAACTCTGGG R: AGAGCTGATTCCGAAGTTGG

##### RNA Interference

Small interfering RNAs (siRNAs) were purchased from Thermo Fisher Scientific as follows: *piezo1* (Assay ID: 502463), *trpv4* (Assay ID: 182204), and scrambled (Silencer negative control no. 1 siRNA, No. AM4611). siRNA with concentration 4 × 10^−9^ m was mixed with Lipofectamine RNAiMAX transfection reagent (No. 13778075, Thermo Fisher Scientific) for 15 min in Opti‐MEM (No. 31985062, Thermo Fisher Scientific). The mixture was then applied to cells and cultured for 3 d. Quantitative PCR was used to confirm the knockdown efficacy of each target gene compared to the scrambled control.

##### Statistical Analysis

Statistical hypothesis tests were performed in GraphPad Prism. Where standard deviations did not vary between test groups, one‐variable analysis was performed using ordinary one‐way ANOVA followed by Tukey's multiple comparison testing. Where standard deviations were variable, one‐variable analysis was performed using one‐way Welch's ANOVA followed by Dunnett T3 multiple comparison testing. For two‐factor analysis, such as cell volume as a function of time, repeated measures two‐way ANOVA with Sidak's multiple comparison testing were used. A *p*‐value less than 0.05 established statistical significance.

## Conflict of Interest

The authors declare no conflict of interest.

## Supporting information

Supporting Information
