# Supplementary material for: Controlled Deposition of 3D Matrices to Direct Single Cell Functions
Source: Adv Sci (Weinh). 2020 Sep 3;7(20):2001066. doi: 10.1002/advs.202001066 (PMC7578851; doi:10.1002/advs.202001066)
Supplement: Supplementary file 1 — Supporting Information [file ADVS-7-2001066-s001.pdf]

© 2020 Wiley-VCH GmbH

ADVANCED SCIENCE
Open Access

# Supporting Information

for *Adv. Sci.,* DOI: 10.1002/advs.202001066

## Controlled Deposition of 3D Matrices to Direct Single Cell Functions

*Sing Wan Wong, Stephen Lenzini, Raymond Bargi, Zhe Feng, Celine Macaraniag, James C. Lee, Zhangli Peng, and Jae-Won Shin**

**Supporting Information**

**Title:** Controlled deposition of three-dimensional matrices to direct single cell functions

**Authors**: Sing Wan Wong[1,2], Stephen Lenzini[1,2], Raymond Bargi[1,2], Zhe Feng[3], Celine Macaraniag[2], James C. Lee[2], Zhangli Peng[2], and Jae-Won Shin[1,2*].

**Affiliations**: [1]Department of Pharmacology and [2]Department of Bioengineering, University of Illinois at Chicago College of Medicine, Chicago, IL 60612, [3]Department of Aerospace and Mechanical Engineering, University of Notre Dame, Notre Dame, IN 46556.

*Correspondence to J-W. S.: shinjw@uic.edu

This file contains:

Supporting Text
Supporting Figures 1-7

**Supporting Text**

**Stress of the gel surrounding an expanding rigid cell**

***1. Analytical solution in the case of linear elasticity (Figure 3A, i and ii)***

Since the problem is spherically symmetric ($u_\theta = u_\varphi = 0$), in the spherical coordinate $(r,\theta,\varphi)$, we have:

$$\nabla \cdot \boldsymbol{u} = \frac{1}{r^2}\frac{d(r^2 u_r)}{dr} = 3a \tag{1}$$

where **u** is the displacement vector, $r$ is the radius between cell surface and gel surface, and $a$ is a constant to be determined. Eq. 1 was derived from the fundamental equation of conservation of linear momentum (Cauchy equation) in the case of spherical symmetry.

Then, by integrating Eq. 1, we have:

$$u_r = ar + \frac{b}{r^2} \tag{2}$$

where $a$, $b$ are constants to be determined by boundary conditions.

The strains are given as:

$$e_{rr} = \frac{\partial u_r}{\partial r} = a - \frac{2b}{r^3} \tag{3}$$

$$e_{\theta\theta} = e_{\phi\phi} = \frac{u_r}{r} = a + \frac{b}{r^3}\,. \tag{4}$$

The stresses are given as:

$$\sigma_{rr} = \lambda 3a + \frac{E}{1+\nu} e_{rr} = \frac{E}{1-2\nu} a - \frac{2E}{1+\nu}\frac{b}{r^3} \tag{5}$$

$$\sigma_{\theta\theta} = \sigma_{\phi\phi} = \lambda 3a + \frac{E}{1+\nu} e_{\theta\theta} = \frac{E}{1-2\nu} a + \frac{E}{1+\nu}\frac{b}{r^3} \tag{6}$$

where $E$ is Young's modulus, $\lambda = \frac{E\nu}{(1+\nu)(1-2\nu)}$ is the Lame constant and $\nu$ is the Poisson's ratio.

Applying the boundary conditions $u_r(r = r_1) = u_0$ and $\sigma_{rr}(r = r_2) = 0$, we have:

$$u_r(r = r_1) = ar_1 + \frac{b}{r_1^2} = u_0 \tag{7}$$

and

$$\sigma_{rr}(r = r_2) = \frac{E}{1-2\nu} a - \frac{2E}{1+\nu}\frac{b}{r_2^3} = 0\,. \tag{8}$$

Solving Eqs. 7 and 8 together, we obtain:

$$a = \frac{2(1-2\nu)b}{(1+\nu)r_2^3} \tag{9}$$

and

$$b = \frac{u_0}{\frac{2(1-2\nu)r_1}{(1+\nu)r_2^3}+\frac{1}{r_1^2}} . \tag{10}$$

Plugging them into Eq. (6), we obtain:

$$\sigma_{\theta\theta} = \sigma_{\phi\phi} = \frac{E}{1-2\nu}a + \frac{E}{1+\nu}\frac{b}{r^3} = \frac{Eu_0 r_1^2}{r_2^3(1+\nu)+2r_1^3(1-2\nu)}\left(2+\frac{r_2^3}{r^3}\right) . \tag{11}$$

At $r = r_1$, the tension stress is given as:

$$\sigma_{\theta\theta}(r=r_1) = \sigma_{\phi\phi}(r=r_1) = \frac{E}{1-2\nu}a + \frac{E}{1+\nu}\frac{b}{r_1^3} = \frac{Eu_0 r_1^2}{r_2^3(1+\nu)+2r_1^3(1-2\nu)}\left(2+\frac{r_2^3}{r_1^3}\right) . \tag{12}$$

Divide the numerator and denominator in Eq. (12) by $r_1^3(1+\nu)$ and rearrange the expression, we have:

$$\sigma_{\theta\theta}(r=r_1) = \frac{Eu_0/[r_1(1+\nu)]}{r_2^3/r_1^3+2(1-2\nu)/(1+\nu)}\left(2+\frac{r_2^3}{r_1^3}\right) = \frac{Eu_0}{r_1(1+\nu)}\left[\frac{6\nu/(1+\nu)}{r_2^3/r_1^3+2(1-2\nu)/(1+\nu)}+1\right] . \tag{13}$$

In terms of gel thickness ($d_{gel}$), Eq. (13) becomes:

$$\sigma_{\theta\theta}(r=r_1) = \frac{Eu_0}{r_1(1+\nu)}\left[\frac{6\nu/(1+\nu)}{(r_1+d_{gel})^3/r_1^3+2(1-2\nu)/(1+\nu)}+1\right] . \tag{14}$$

Since $0 \le \nu \le 0.5$, with fixed values of $r_1$ and $u_0$, the tension stress $\sigma_{\theta\theta}$ increases with decreasing $d_{gel}$. In other words, the tension increases with thinner gels, regardless of ν.

When the material is incompressible, i.e. Poisson's ratio $\nu = 0.5$, we have:

$$\sigma_{\theta\theta}(r=r_1) = \frac{Eu_0}{r_1}\left(\frac{4r_1^3}{3(r_1+d_{gel})^3}+\frac{2}{3}\right) . \tag{15}$$

When $r_2 = \infty$, i.e. a bulk gel, the minimum tension stress is given by:

$$\sigma_{\theta\theta_{\min}} = \frac{2Eu_0}{3r_1} = 192.95 \text{ Pa} . \tag{16}$$

In addition, the volume strain is given as:

$$e_v = e_{rr} + e_{\theta\theta} + e_{\phi\phi} = 3a = \frac{6(1-2\nu)b}{(1+\nu)r_2^3} = \frac{u_0\frac{6(1-2\nu)}{(1+\nu)r_2^3}}{\frac{2(1-2\nu)r_1}{(1+\nu)r_2^3}+\frac{1}{r_1^2}} = \frac{6u_0(1-2\nu)}{2(1-2\nu)r_1+\frac{(1+\nu)r_2^3}{r_1^2}}. \quad (17)$$

Since $0 \leq \nu \leq 0.5$, $e_v$ is constant and positive everywhere—i.e. the volume is expanded and the polymer density is decreased with the same amount everywhere. In other words, if $\nu < 0.5$, gel volume is expected to expand as a result of cell volume expansion.

***2. Finite element solution in the case of large deformation with rubberlike elasticity (Figure 3A, iii)***

In the case of large deformation with rubber-like elasticity, no analytical solution is available. We applied nonlinear finite element method to solve the boundary value problem using the commercial finite element package Abaqus. Since the problem is axisymmetric, we used the axisymmetric formulation to solve the 3D problem. Approximately 2000 quadrilateral axisymmetric elements were used, and the convergence was reached at such resolution. We applied the displacement boundary condition ($u = u_0$) at the inner boundary, and stress-free boundary condition ($\sigma_{rr}(r = r_2) = 0$) at the outer boundary. We used the incompressible neo-Hookean material to consider the rubber-like elasticity of the gel. The strain energy potential of neo-Hookean material is given as

$$U = \frac{G}{2}(I_1 - 3) \quad (18)$$

where $I_1 = \lambda_1^2 + \lambda_2^2 + \lambda_3^2$ is the first invariant of deformation, $\lambda_1, \lambda_2, \lambda_3$ are the principal stretches, and $G$ is the shear modulus.

We considered three different cases with varied gel thicknesses (5, 15, 100 $\mu m$). We used 100 $\mu m$ to approximate the bulk gel case. The cell (the inner sphere) has an initial radius of 7.82 $\mu m$ and expands its volume by 50%. The incompressible neo-Hookean hyperelastic material with a Young's modulus of 2000 Pa was used for the gel. The results show that the maximum tension stress near the inner surface decreases with increased gel diameters. It is consistent with the analytical solution in Eq. 15.

To compare the finite element solution with the analytical solution in Eq. 15, let $E = 3G = 2000$ Pa, $u_0 = (1.5^{1/3} - 1)\cdot 7.82\ \mu m = 1.13\ \mu m$, $r_1 = 7.82\ \mu m$.

For $r_2 = (12.82, 22.82, 107.82)\ \mu m$, the analytical solution Eq. 15 gives:

$$\sigma_{\theta\theta}(r = r_1) = 280.5, 208.5, 193.1\ Pa,$$

while the finite element simulation gives

$$\sigma_{\theta\theta}(r = r_1) = 258.3, 182.3, 164.2\ Pa.$$

## Supporting Figures

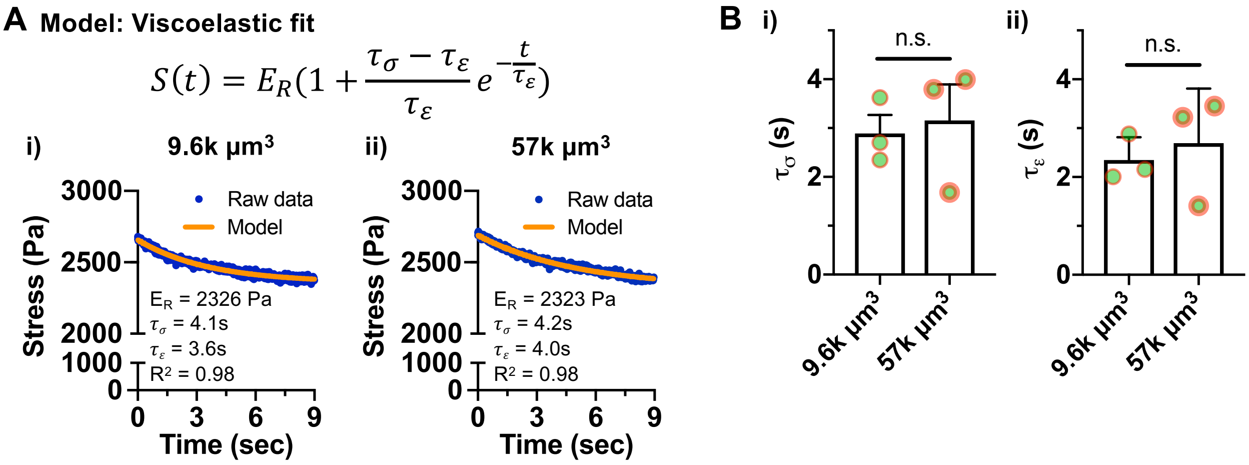


**Figure S1. Characterization of stress relaxation with varied gel deposition. (A)** The viscoelastic properties of the gel coating were extracted from the AFM data by fitting to the stress relaxation model (see Experimental Section). Representative graphs showing stress over time under a 9-second dwell for **(i)** thin (9600 µm$^3$) and **(ii)** thick (57,000 µm$^3$) gels that encapsulate single mouse MSCs. Blue dots indicate raw data and yellow lines indicate curve fits. For each graph, relaxed modulus ($E_R$), time of relaxation of deformation under constant load ($\tau_\sigma$), time of relaxation of load under constant deformation ($\tau_\varepsilon$), and $R^2$ values from data fitting are shown. **(B)** Quantification of **(i)** time of relaxation of deformation under constant load ($\tau_\sigma$) and **(ii)** time of relaxation of load under constant deformation ($\tau_\varepsilon$). The data are from $n = 3$ independent experiments and shown as mean ± S.D. Each data point is mean of at least 5 gels per experiment.

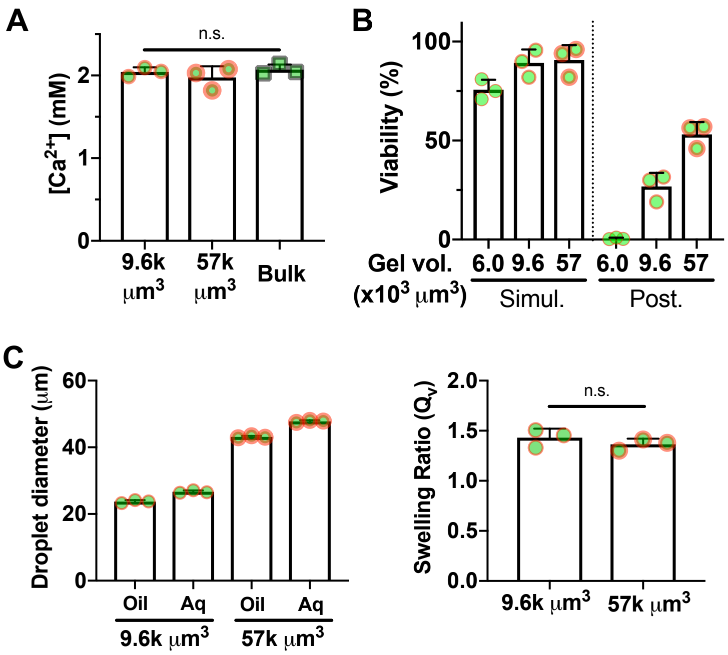


**Figure S2. Characterization of the controlled gel deposition method. (A)** Free [$Ca^{2+}$] in the media after 3-hour culture at 37°C. **(B)** Cell viability as a function of varied gel deposition with simultaneous (simul.) or post-emulsion (post.) crosslinking. **(C)** Quantification of alginate microgel swelling. (Left) Droplet diameters in emulsion *vs.* after swelling in aqueous buffer for 3 hours at 37°C with varied alginate gel volume (Right). Estimation of swelling ratios ($Q_v$) between droplet volume after swelling *vs.* in emulsion. Assuming spherical morphology, $Q_v$ = (radius of microgels after aqueous extraction)$^3$/(radius of droplets in the emulsion)$^3$. All data are from $n = 3$ independent experiments and shown as mean ± S.D. For (A) and (B) each data point is mean of technical duplicates, while for (C), each data point is mean of 10 droplets.

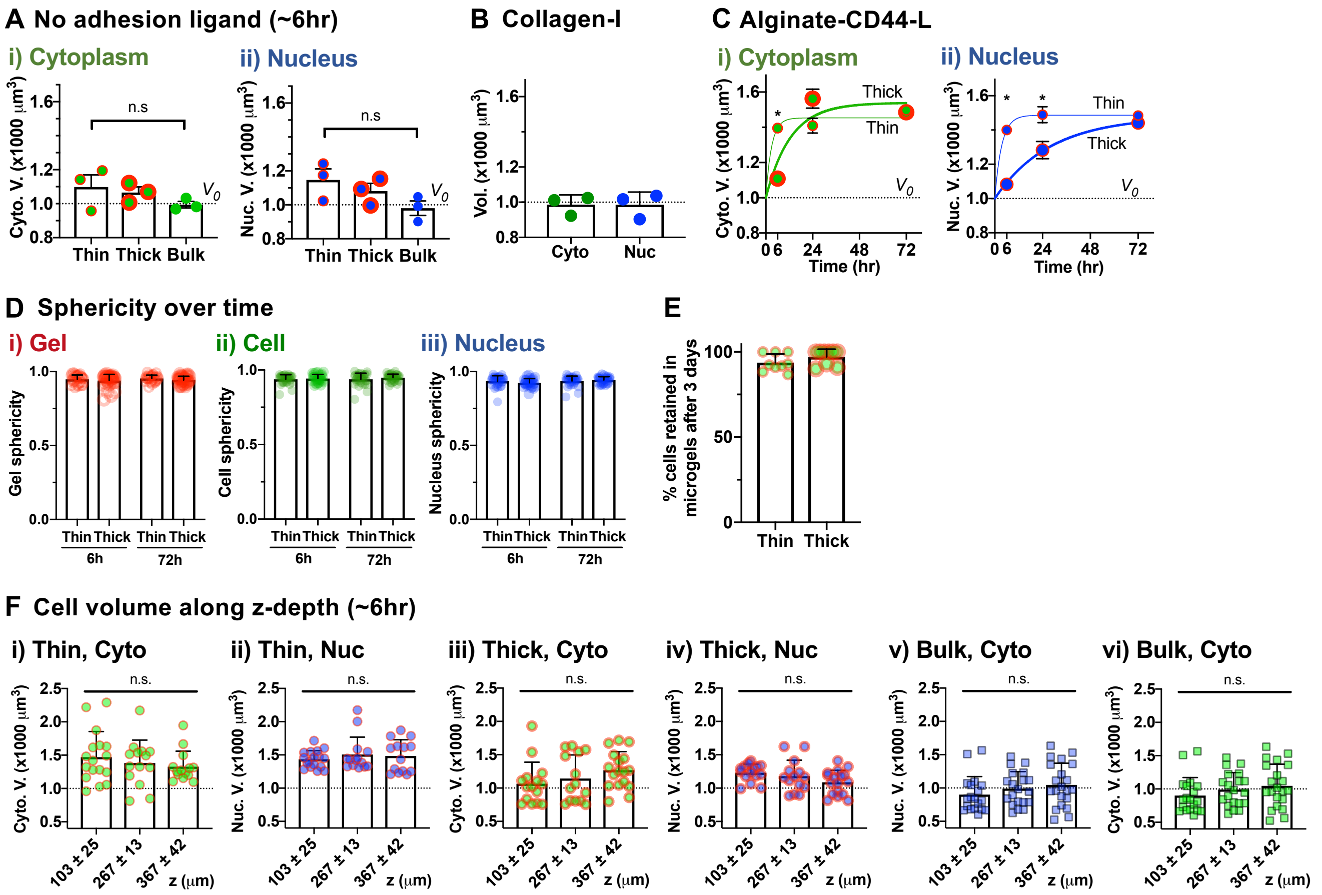


**Figure S3. Characterization of single cell volume expansion with varied gel deposition. (A)** Quantification of (i) cytoplasmic and (ii) nuclear volumes at 6 hours after encapsulation in alginate gels without an adhesion ligand. Mean ± S.D. from *n* = 3 independent experiments, 15 cells per experiment. **(B)** Cytoplasmic and nuclear volumes of MSCs after embedding in collagen-I gel (1.25 mg/ml) for 2 hours. Mean ± S.D. from *n* = 3 independent experiments, 15 cells per experiment. **(C)** Volume expansion kinetics of (i) cytoplasm and (ii) nucleus after encapsulation of MSCs in the thin or thick alginate gel conjugated to a CD44-binding peptide ('CD44-L', A5G27). The data points were fit to one-phase exponential association equation: $V = V_0 + (V_m - V_0)(1 - e^{-kx})$, where $V_0 = 1000\ \mu m^3$. ($V_m$ ($\mu m^3$), $t_{1/2}$ (= ln2/k, h)) values for each group - thin: cytoplasm (1453, 2.03), nucleus (1487, 2.41); thick: cytoplasm (1539, 12.57), nucleus (1479, 19.22). All data are from *n* = 4 independent experiments, 15 cells per experiment, and shown as mean ± S.E.M. *, (i) $p = 0.047$ (ii) $p = 0.0032$ for column factor (varied gel deposition) via two-way ANOVA comparisons followed by Sidak's multiple comparisons test. **(D)** Sphericity of (i) gels, (ii) cells, and (iii) nuclei at 6 or 72 hours after encapsulation in the thin or thick alginate-RGD gel. *n* = 34 pooled from 3 independent experiments, and shown as mean ± S.D. **(E)** Percentage of MSCs retained within the thin or thick alginate-RGD gel after 3-day culture. The data are from *n* = 10 images pooled from 2 independent experiments, and shown as mean ± S.D. **(F)** Measurement of cell volume sampled at three different gel heights (range: 0-450 μm), 6 hours after single cell encapsulation in alginate gels followed by collagen-I gel embedding for thin and thick alginate gel-coated cells: (i) Thin gel, cytoplasmic volume, (ii) Thin gel, nuclear volume, (iii) Thick gel, cytoplasmic volume, (iv) Thick gel, nuclear volume, (v) Bulk gel, cytoplasmic volume, (vi) Bulk gel, nuclear volume. The data are $n \geq 14$ cells pooled from 3 independent experiments, and shown as mean ± S.D.

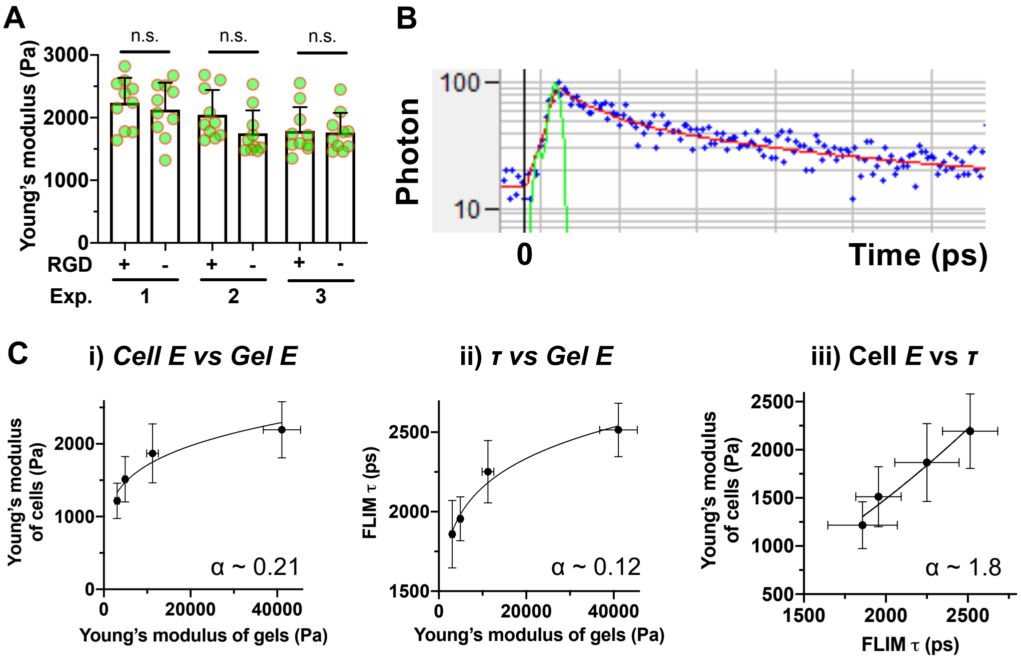


**Figure S4. Characterization of gel mechanics and cell membrane tension. (A)** Young's modulus (*E*) of the thin gel with or without RGD measured at ~8 hours after encapsulation of single MSCs. The data are from $n = 3$ independent experiments, 10 gels per experiment, and shown as mean ± S.D. S.D. of each bar graph ranges from 17.8% to 21.3% of mean. *E* of the thin gel is not statistically different between with and without RGD in each experiment as assessed by two-tailed T-test. **(B)** A representative plot showing fluorescence decay of Flipper-TR in cells over time. Blue = data from a mouse MSC, 2 hours after embedding in a collagen-I gel; Red = a two-phase exponential decay fit; Green = system response. **(C)** Correlation analyses of MSCs cultured on 2D PEGDA-RGD gels showing (i) Young's modulus (*E*) of cells vs. *E* of gels, (ii) FLIM decay time (τ) vs. *E* of gels, (iii) *E* of cells vs. FLIM τ. The data points from each graph were fitted to the power-law equation $Y \sim X^{\alpha}$, where $\alpha$ is indicated in each graph. The data are from $n = 10$ cells or gel measurements in each group, and shown as mean ± S.D.

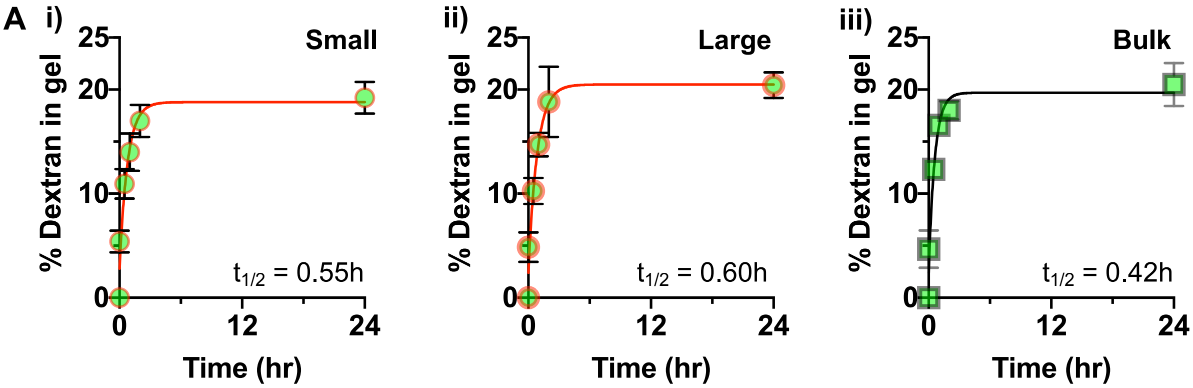


**Figure S5. Characterization of diffusion into alginate gels. (A)** Diffusion kinetics of FITC-Dextran (~20 kDa) into **(i)** small microgels (~25 μm diameter, equivalent to the size of thin gel-coated MSCs), **(ii)** large microgels (~45 μm diameter, equivalent to the size thick gel-coated MSCs), and **(iii)** bulk alginate gels. The data points from each graph were fit to the one-phase exponential association equation: % FITC-dextran in gel = plateau$\cdot$(1 - $e^{-kt}$). (plateau (%), $t_{1/2}$ (= ln2/k, h)) values for each group - small (18.8, 0.55), large (20.5, 0.60), bulk (19.7, 0.42). The data are from $n$ = 3 independent experiments, and shown as mean ± S.D.

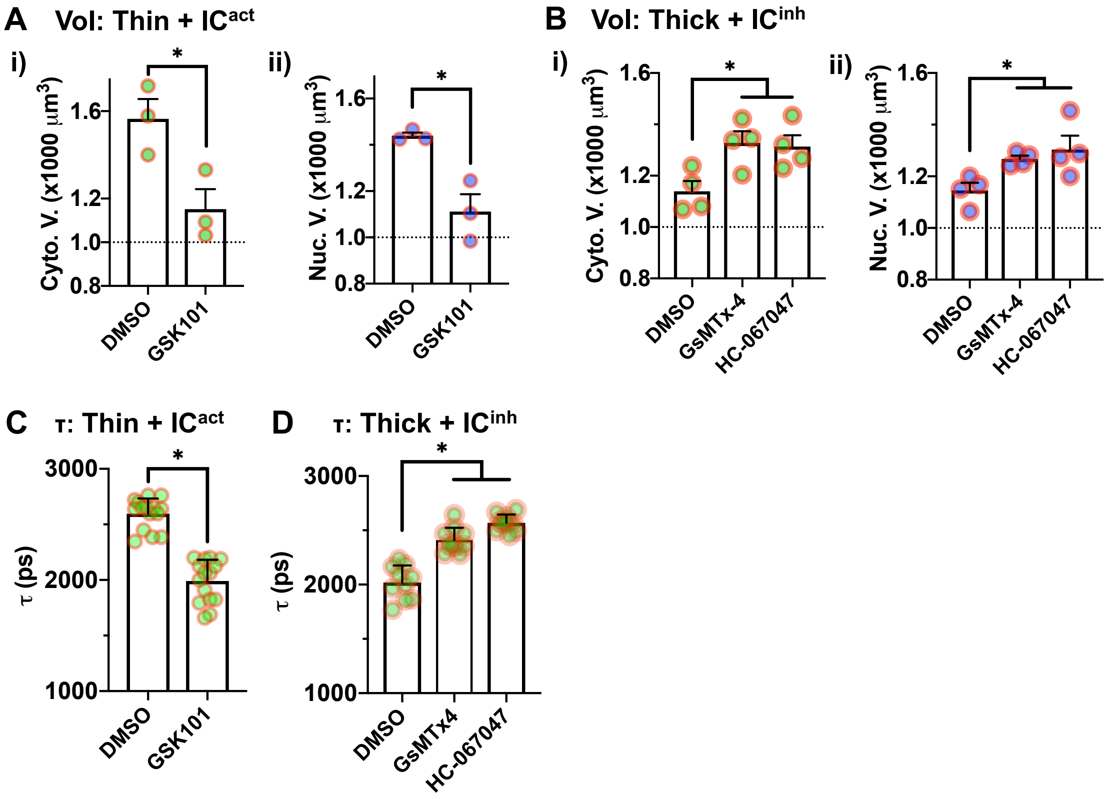


**Figure S6. Effects of ion channel modulators on cell volume and membrane tension with varied gel deposition. (A)** Quantification of (i) cytoplasmic and (ii) nuclear volumes in the thin alginate-RGD gel at 6 hours after encapsulation, followed by treatment with GSK101 (10 nM) for 2 hours. The data are from $n = 3$ independent experiments, 15 cells per experiment, and shown as mean ± S.E.M. *, (i) $p = 0.0079$, (ii) $p = 0.036$ via paired T-test. **(B)** Quantification of (i) cytoplasmic and (ii) nuclear volumes in the thick alginate-RGD gel at 6 hours after encapsulation, followed by treatment with ion channel inhibitors for 2 hours, including GsMTx-4 (2.5 µM) and HC-067047 (2.5 µM). The data are from $n = 4$ independent experiments, 15 cells per experiment, and shown as mean ± S.E.M. *, (i) $p = 0.003$, (ii) $p = 0.025$ via repeated measures one-way ANOVA, followed by Tukey's multiple comparisons test. **(C)** Quantification of membrane tension (τ) in the thin alginate-RGD gel in response to the TRPV4 activator GSK101. The data are from $n = 15$ cells pooled from 3 independent experiments, and shown as mean ± S.D. *, $p = 1.1 \times 10^{-10}$ via unpaired T-test. **(D)** Quantification of membrane tension (τ) in the thick alginate-RGD gel in response to the indicated ion channel inhibitors. The data are from $n = 10$ cells pooled from 3 independent experiments, and shown as mean ± S.D. *, $p = 1.1 \times 10^{-7}$ via one-way Welch's ANOVA, followed by Dunnett T3 multiple comparisons test.

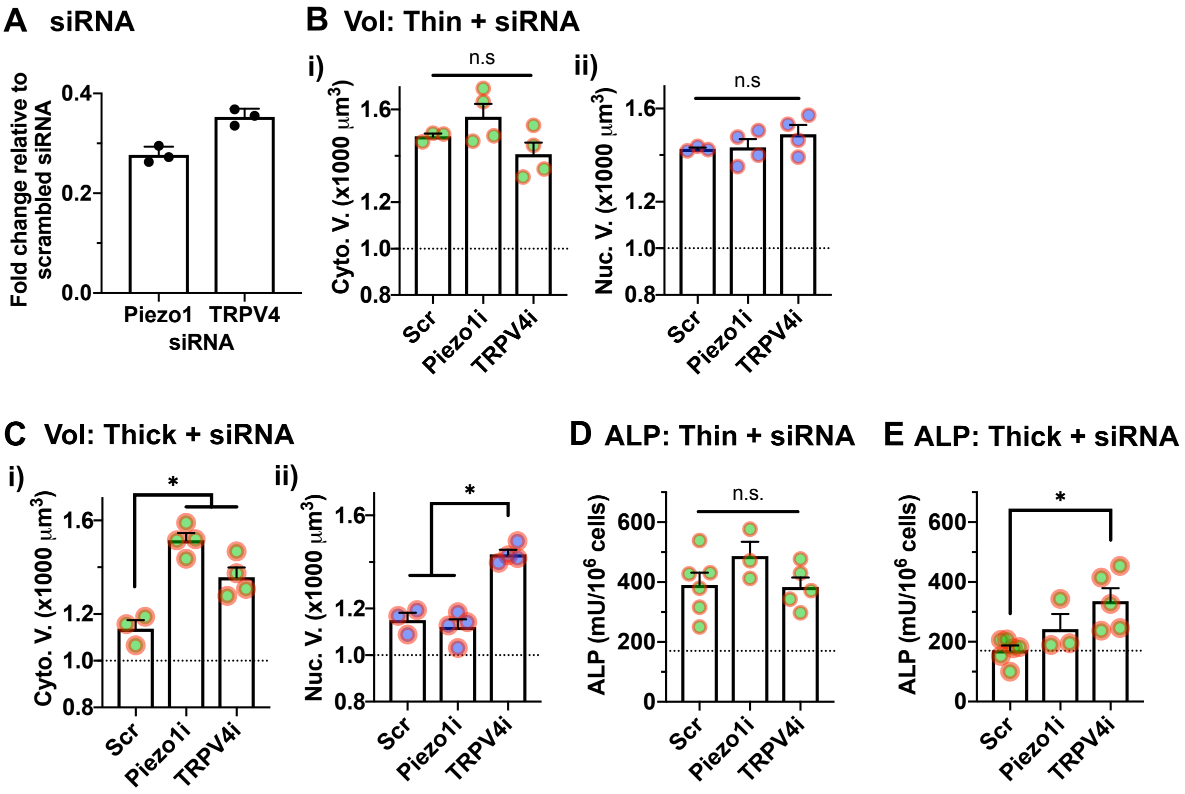


**Figure S7. Effects of siRNA against ion channels on cell volume and osteogenic differentiation of MSCs with varied gel deposition. (A)** siRNA knockdown efficiency of *piezo1* and *trpv4* in MSCs relative to scrambled control after 3-day treatment by transfection. The data are from $n$ = 3 independent experiments, shown as mean ± S.D. **(B)** Effects of siRNA against ion channels on (i) cytoplasmic and (ii) nuclear volumes in the thin alginate-RGD gel. **(C)** Roles of ion channels in (i) cytoplasmic and (ii) nuclear volumes in the thick alginate-RGD gel. *, (i) $p = 0.00044$, (ii) $p = 7.5 \times 10^{-5}$ via ordinary one-way ANOVA, followed by Tukey's multiple comparisons test. For (B) and (C), $n$ = 3 independent experiments for scrambled siRNA control (Scr) and 4 for each of Piezo1 siRNA ($Piezo1_i$) and TRPV4 siRNA ($TRPV4_i$), 15 cells per experiment. **(D)** Quantification of ALP activity in the thin gel after 7-day osteogenic culture of MSCs treated with siRNA. **(E)** Quantification of ALP activity in the thick gel encapsulating siRNA-treated MSCs. $p = 0.013$ via ordinary one-way ANOVA, followed by Tukey's multiple comparisons test. For (D) and (E), $n$ = 6 independent experiments for Scr, $n$ = 5 for $TRPV4_i$, $n$ = 3 for $Piezo1_i$.
